# Brain cortex activity of patients with disorders of consciousness under familiar and unfamiliar voice of subject's own name: an fNIRS-based study

**DOI:** 10.3389/fneur.2026.1789921

**Published:** 2026-06-04

**Authors:** Jinqin Zhang, Zhihong Jiao, Fubiao Huang, Xinping Wang, Xindiao Zhou, Qing Li, Ying Zhang, Liping Long, Yinuo Su, Kexin Cao, Yiheng Han

**Affiliations:** 1 Department of Occupational Therapy, China Rehabilitation Research Center, Feng Tai, Beijing, China; 2 Faculty of Rehabilitation, Capital Medical University, Feng Tai, Beijing, China; 3 Otawara Campus, International University of Health and Welfare, Otawara, Japan; 4 Narita Campus, International University of Health and Welfare, Tokyo, Japan; 5 Graduate School Office, Jilin Sport University, Changchun, Jilin, China; 6 School of Rehabilitation Medicine, Shandong Second Medical University, Weifang, Shandong, China

**Keywords:** auditory sensory stimulation, disorders of consciousness, functional near-infrared spectroscopy, occupational therapy, subject's own name

## Abstract

**Background:**

Although auditory sensory stimulation has been widely used in clinical occupational therapy practice for DOC, there is significant heterogeneity in specific implementation protocols. However, none of these studies have focused specifically on exploring the familiar voice of subject's own name (FV SON) vs. unfamiliar voice of subject's own name (UFV SON) calling. Functional near-infrared spectroscopy (fNIRS) has shown great potential in the clinical assessment of DOC, as it features both high portability and relatively high spatial resolution.

**Objective:**

This study used fNIRS to investigate the real-time cortical activation elicited by auditory sensory stimulation through FV SON and UFV SON in patients with DOC, aiming to provide meaningful guidance for clinical occupational therapy and treatment evaluation.

**Methods:**

A total of 64 participants with DOC were recruited for this study. FNIRS was used to detected blood oxygen signals during FV SON and UFV SON in the subjects. Statistical analyses were performed for intra-group differences and the relationships between fNIRS metrics and the scores of the CRS-*R*.

**Results:**

Participants with DOC under UFV SON stimulation exhibited deactivation in the Left Dorsolateral Pre-frontal Cortex (DLPFC-L). However, compared with the rest state (RS), participants with DOC under FV SON stimulation did not show deactivation in the DLPFC-L. Compared with the UFV SON stimulation, the peak value of participants with DOC under FV SON stimulation was greater in the DLPFC-L. However, compared with the FV SON stimulation, the peak value of participants with DOC under UFV SON stimulation was greater for the TC-L. Compared with the UFV SON stimulation, the initial slope of participants with DOC under FV SON stimulation was greater for PreM & SMC-L. Under FV SON stimulation, the peak values of the DLPFC-L and TC-L were positively correlated with the CRS-*R* VFS score. Under UFV SON stimulation, the peak value of the TC-L was positively correlated with the CRS-*R* total score, CRS-*R* VFS score and CRS-*R* AS score; the peak value of the PMC-L was positively correlated with the CRS-*R* total score, CRS-*R* AFC score and CRS-*R* VFS score; and the peak value of the Broca-L was positively correlated with the CRS-*R* total score, CRS-*R* VFS score and CRS-*R* AS score. Under UFV SON stimulation, the initial slope of the DLPFC-L was positively correlated with the CRS-*R* AFC score.

**Conclusion:**

In conclusion, DOC patients show distinct cortical activation patterns under familiar vs. unfamiliar SON calling stimulation. This study is helpful for guiding auditory stimulation strategies or personalized rehabilitation in DOC. These conclusions warrant further validation in future larger, multicenter studies, multimodal neuroimaging, studies, and intervention-based studies. [ChiCTR2300074202].

## 1 Introduction

Disorders of consciousness (DOC) are pathological alterations in states of consciousness resulting from structural or functional damage to the neural networks governing arousal and awareness. These disorders include coma, the vegetative state [VS; also known as unresponsive wakefulness syndrome (UWS)], and the minimally conscious state (MCS), all of which are defined by standardized diagnostic criteria (1–3, 65), Giacino et al., 2002, (4). A recent addition to the diagnostic classification scheme of patients with DOC is the concept of cognitive motor dissociation (CMD) (5), also known as covert consciousness. Previous research has indicated that the incidence of VS/UWS is approximately 525 cases per million population (PMP) in various countries. Previous research has also suggested that the prevalence of MCS is tenfold greater than that of VS (66) DOC significantly affects patient survival rates, ability to perform activities of daily living (ADL), quality of life, employment, and relationships postinjury, resulting in considerable caregiving demands and substantial societal medical resource expenditures. Treatment goals may vary over the course of recovery, with the end goal of improving the occupational performance, participation, and quality of life of patients with DOC.

While pharmacological agents and neuromodulatory interventions remain the cornerstone treatments, non-invasive approaches such as sensory stimulation have emerged as complementary therapeutic modalities. A longstanding theoretical perspective suggests that regulated sensory stimulation may facilitate positive therapeutic outcomes; however, patients exposed to undifferentiated bombardment of sensory information lose information processing ability owing to habituation (6). Rehabilitation therapists, who are proficient in sensory modulation principles, can provide controlled sensory stimuli calibrated to higher thresholds of reticular neurons, thereby increasing cortical activity (7). Auditory sensory stimulation is more critical than other sensory stimuli (e.g., visual, verbal, and motor functions) for the following reasons: auditory function is the last perceptual function that coma patients lose (8) and auditory sensory stimulation is simple to implement, requires no active cooperation from patients, and is applicable to various types of patients with DOC.

Many scientific studies have utilized electroencephalography (EEG) and functional magnetic resonance imaging (fMRI) to investigate in depth the neural activity and activation patterns of the brain under different auditory sensory stimulation paradigms, aiming to elucidate the central mechanisms of auditory sensory stimulation in rehabilitation therapy. Moreover, these findings have contributed to the establishment of diagnostic differentiation and prognostic evaluation standards for disorders of consciousness (9–12). In particular, although traditional EEG technology has advantages such as high temporal resolution and rapid response, its relatively low spatial resolution makes it difficult to precisely characterize fine-grained activation patterns of distinct cortical areas related to consciousness. In contrast, while fMRI offers superior spatial resolution, its applicability for convenient real-time bedside assessments of patients with disorders of consciousness is limited. Given this background, functional near-infrared spectroscopy (fNIRS) has shown great potential in the clinical assessment of DOC, as it features both high portability and relatively high spatial resolution (13–16). Current studies using fNIRS have investigated the effects of acupuncture, motor imagery, and auditory sensory stimulation on cortical activation in patients with DOC (17, 18).

Although auditory sensory stimulation has been widely used in clinical occupational therapy practice, there is significant heterogeneity in specific implementation protocols. Current intervention studies involving auditory sensory stimulation include mainly subject's own name (SON) calling by family members, SON calling by nurses, natural environmental sounds, preferred music, and reminiscence stories from before the onset of illness. Existing randomized controlled studies have explored the rehabilitation efficacy of different types of auditory sensory stimulation for patients with DOC; however, clinical evidence regarding their therapeutic effects remains inconsistent (11, 19, 20, 67). Moreover, none of them have examined how auditory sensory stimulation through SON calling with different levels of familiarity affects cortical activation in patients with DOC. As a result, the underlying mechanisms remain unclear.

It is of great significance to explore the effects of auditory stimuli with different familiarity on patients with DOC. Previous studies have shown that the dorsolateral pre-frontal cortex (DLPFC), frontal pole area (FPA), frontal eye field (FEF), temporal cortex (TC), pre-motor and supplementary motor cortex (PreM & SMC), and somatosensory cortex (SSC), as key nodes of neural networks such as the executive-control network (ECN), the default-mode network (DMN), and the salience network (SN), are closely associated with consciousness (21, 22). Previous studies have demonstrated that familiar and unfamiliar sound stimuli exert distinct effects on three neural networks and key nodes, which may constitute the underlying mechanism for improving DOC (23–27).

In this study, fNIRS was used to investigate the real-time cortical activation elicited by auditory sensory stimulation through FV SON calling and UFV SON calling in patients with DOC. These findings are expected to provide meaningful guidance for clinical occupational therapy and treatment evaluation and to serve as a reference for future applications of fNIRS testing based on SON calling auditory paradigms in the differential diagnosis and prognostic assessment of disorders of consciousness. Furthermore, this study further explored the correlation between real-time cortical activation and the degree of DOC.

## 2 Methods

### 2.1 Participants

From September 2024 to September 2025, patients with DOC were recruited from the Department of Intensive Rehabilitation and the Department of Neurosurgery at the China Rehabilitation Research Center. The study protocol was approved by the Ethics Committee of the China Rehabilitation Research Center (Registration No.: ChiCTR2300074202), and written informed consent was obtained from the relatives of each participant prior to eligibility assessment. No adverse events occurred during the study period. This study was supported by the Youth Research Fund of the China Rehabilitation Research Center (Grant No.: 2023ZX-Q11) and the Health Commission of Fengtai District, Beijing (Grant No.: 2025-022).

#### 2.1.1 Inclusion criteria

(1) Patients meeting the prolonged Disorders of Consciousness (pDOC) diagnostic criteria of the European Academy of Neurology guidelines (more than 4 weeks after brain injury);
(2) Patients were diagnosed with a coma, UWS/VS, or MCS on the basis of five Coma Recovery Scale–Revised (CRS-*R*) assessments within 1 week;
(3) Definite etiology and stable vital signs;
(4) Aged 18–75 years;
(5) Right-handed;
(6) No participation in other clinical trials within the past 3 months;
(7) Written informed consent provided by a family member or a legally authorized representative.

#### 2.1.2 Exclusion criteria

(1) Clinically unstable condition; severe and uncontrolled respiratory or circulatory complications; acute onset stage; status epilepticus; multiple trauma; limb fractures; large-area skin defects; progressive deterioration of the condition; or uncontrolled active intracranial hemorrhage;
(2) History of psychiatric disorders, drug abuse, or chronic alcohol addiction;
(3) Presence of intracranial metal implants, intracranial tumors, intracranial infections, or any clinically significant or unstable medical condition;
(4) Active infection;
(5) Cranial defects or abnormalities preventing valid fNIRS data acquisition;
(6) Severe hearing impairment;
(7) Individuals with exceptionally high musical expertise or previous professional engagement in music-related occupations.

#### 2.1.3 Withdrawal and termination criteria

Participants who were unable to tolerate the study protocol or whose clinical condition changes during the study were withdrawn.

### 2.2 Blinding design

Owing to the study design and the overt symptoms of patients with DOC, implementing full blinding is challenging and not entirely feasible. However, standard operating procedure training was conducted prior to study initiation, and the following measures were implemented to minimize potential biases and enhance study validity.

(1) Assessment: the researchers who conducted fNIRS data collection and behavioral assessments were blinded to the study hypotheses and the specific objectives related to the testing. They adhered to standardized assessment protocols to ensure consistency across evaluations.
(2) Data analysis: the researchers responsible for analyzing the fNIRS data were blinded to the type of auditory sensory stimulation and demographic information of the participants during data pre-processing and initial analyses. Group assignments were encoded numerically to prevent bias in data interpretation.
(3) Study participants: participants with DOC were unaware of the study objectives because of their impaired consciousness, thereby minimizing potential bias from the participant side.

### 2.3 Behavioral evaluation

For baseline assessments, the CRS-*R* were administered independently by two trained evaluators in a randomized order.

**CRS-*R***: one evaluator independently conducted CRS-*R* assessments for each patient over five consecutive days in a randomized order, and the best performance across these assessments was selected as the final score. The Chinese version of the CRS-*R* is a reliable and sensitive diagnostic tool that effectively differentiates UWS and MCS. The internal consistency, test–retest reliability, interrater reliability, and diagnostic validity of the Chinese CRS-*R* are good (28). The scale evaluates six functional domain subscales: auditory function scale (AFS), visual function scale (VFS), motor function scale (MFS), oromotor function scale (OFS), communication scale (CS), and arousal scale (AS).

### 2.4 fNIRS data acquisition

#### 2.4.1 fNIRS testing

**Figure 1:**
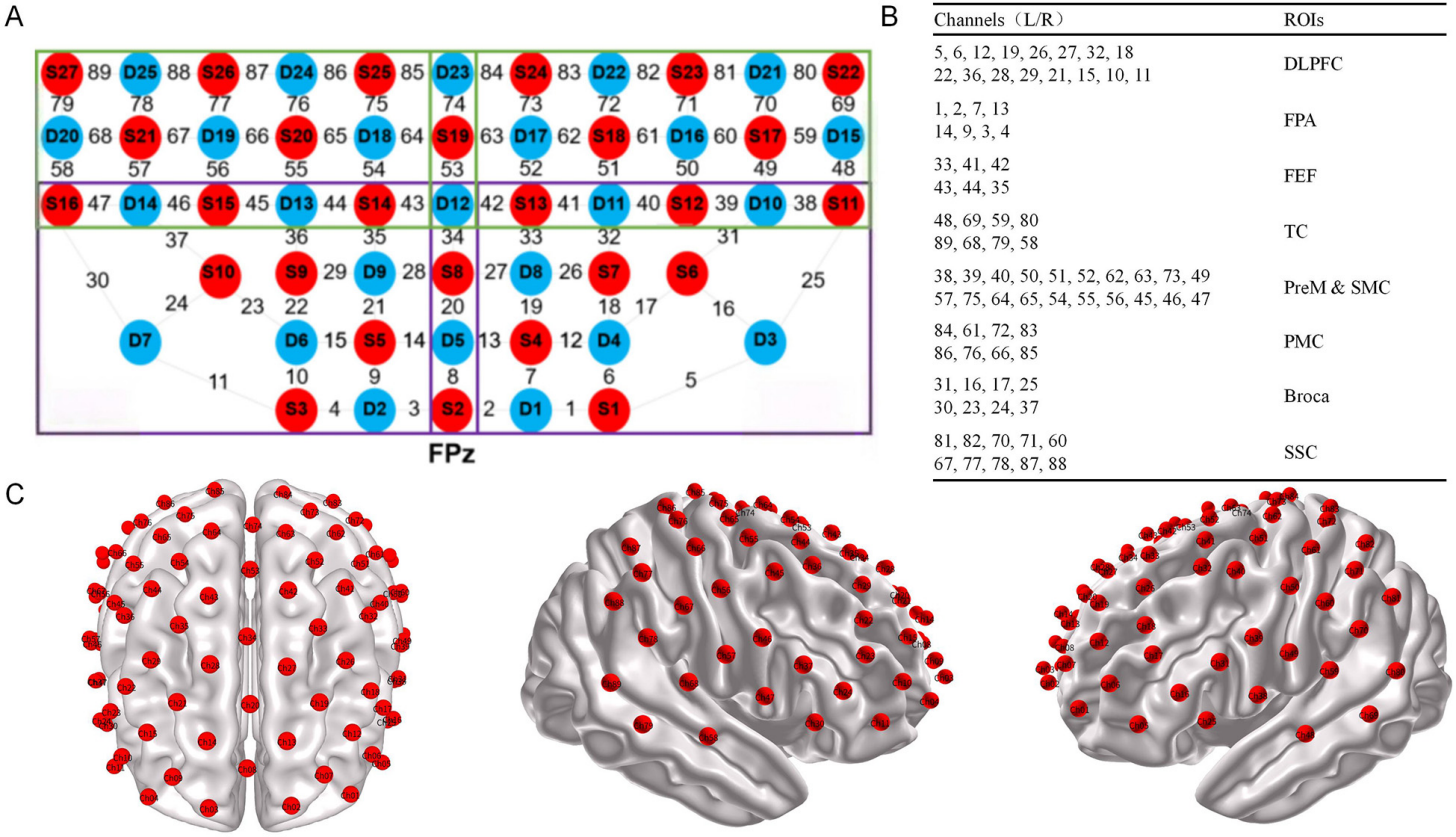
**(A)** 2D layout of optodes.Red circles represent photon emitters, blue circles represent photon detectors. **(B)** Assignment of 89 Channels to 16 ROIs, corresponding to 8 anatomically symmetrical brain areas: dorsolateral prefrontal cortex (DLPFC), frontal pole area (FPA), frontal eye field (FEF), temporal cortex (TC), premotor and supplementary motor cortex (PreM & SMC), primary motor cortex (PMC), somatosensory cortex (SSC) and Broca's area. **(C)** 3D spatial registration of 89 fNIRS channels. Red circles represent fNIRS channels.

Resting-state fNIRS measurements were performed using the BrainScope system (Wuhan Znion Technology Co., Wuhan, China). The system employs dual-wavelength laser diodes (690 nm and 830 nm) with a sampling rate of 20 Hz. A total of 27 emitters and 25 detectors were configured, resulting in the formation of 89 channels. Each channel was defined by a source–detector pair spaced 3 cm apart (Figures 1A, B).

The optode arrangement followed the internationally recognized 10–20 system, with source S2 positioned at the Fpz point. Spatial coordinates of reference points (Nz, Cz, AL, and RL) and all optodes were recorded using a 3D digitizer (NirMap, Wuhan Zilian Hongkang Technology Co., Wuhan, China). The channel locations were determined on the basis of the reference points and optode coordinates (29). On the basis of NFRI registration, the probabilities of obtaining the Montreal Neurological Institute (MNI) coordinates and the Brodmann area (BA) of each measurement channel were obtained Supplementary Table S1) and subsequently classified into eight Reigns of Interest (ROIs; Figure 1C).

The evaluators brought the fNIRS system to the patient's bedside to conduct testing. Participants were positioned lying in bed, while the evaluators turned off the room lights and drew the curtains. The fNIRS helmet and headphones were then fitted onto the patients. Before collection, the participant was fitted with a fiber optic cap, and the positions of the emitter probes and detector probes were adjusted. The hair was carefully ruffled to ensure that the probes were in close contact with the subject's skin to obtain better signal quality. Before each collection, a signal gain check was performed to ensure the quality of data collection and that all the channels showed good signals. The E-Prime program was used to synchronize auditory sensory stimulation presentation with fNIRS data acquisition. Auditory stimuli, including FV SON calling and UFV SON calling, were presented in a randomized order. For the fNIRS assessment, two trained evaluators conducted the testing.

FV SON calling auditory sensory stimulation: after obtaining written informed consent from the participants' relatives, researchers recorded name-calling audio from individuals familiar to the participants. The audio content consisted of the phrase “Hello, [SON].” A “familiar” person was defined as someone who (1) had known the participant for at least 1 year prior to the injury, and (2) had engaged in daily interactions with the participant for at least 1 year before the DOC. Researchers instructed familiar individuals to speak in a normal voice (volume and pitch) using typical speech patterns (prosody, intonation, and rate) without altering their natural pronunciation. The audio recording was processed using the sound editing software Audacity to adjust its duration and quality. Interfering background noise, such as echoes, pops, or other white noise, was removed, while the speech pattern (e.g., rate) remained unchanged. The volume was adjusted to a range of 50–75 dB using the same software. The processed audio files were then loaded onto a computer playback system, and researchers verified the audio through headphones to ensure a comfortable listening level without abrupt volume changes.

UFV SON Calling Auditory Sensory Stimulation: UFV SON calling audio for each participant was created using AI software (iFlytek, China). The UFV SON calling audio was matched to the FV SON calling audio in terms of volume, pitch, prosody, intonation, and speech rate.

**Figure 2:**
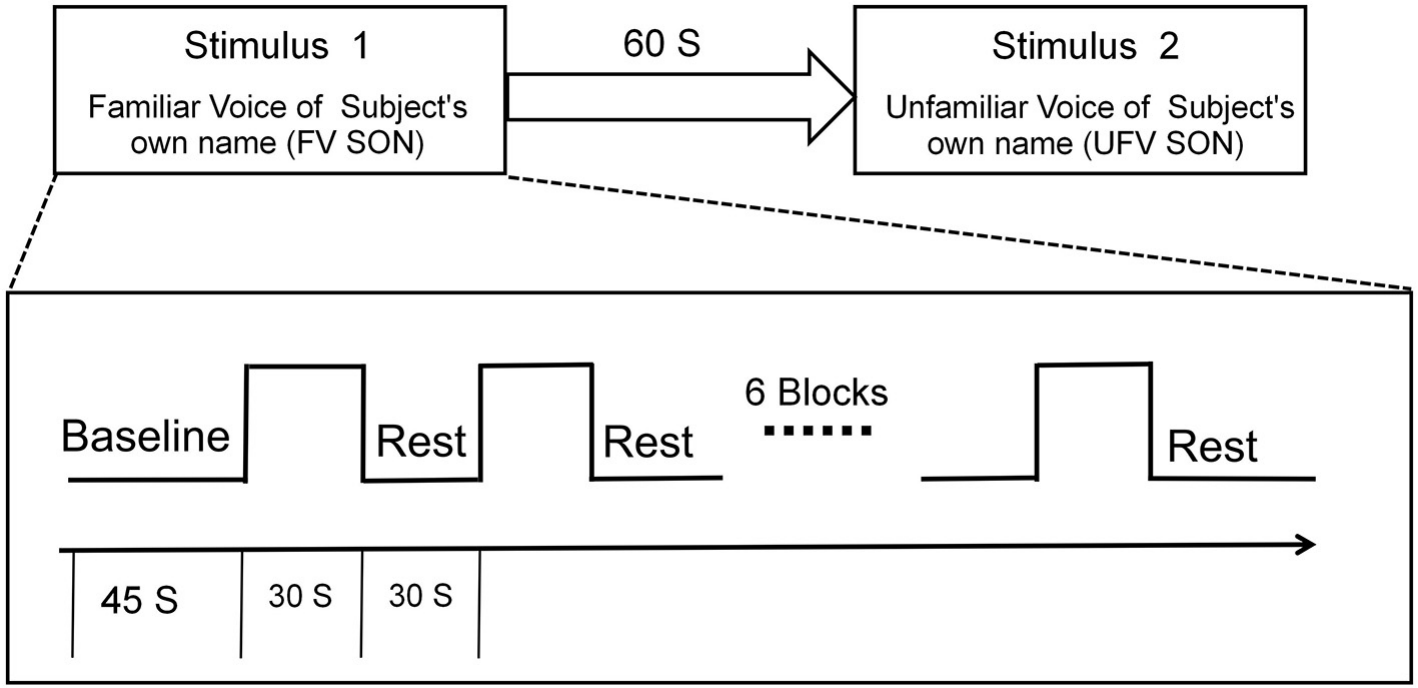
fNIRS detection protocol (SON stimulation paradigm).

Auditory sensory stimulation paradigm: each SON calling auditory stimulus lasted 7 min, including 30-s baseline periods at the beginning and end. The paradigm consisted of six auditory stimulation blocks, each lasting 60 s, comprising 30 s of name calling followed by a 30-s rest interval (Figure 2).

#### 2.4.2 fNIRS data processing

fNIRS data were processed using MATLAB (2024a) and NirMaster (Wuhan Znion Technology Co., Wuhan, China) (S. (30)). The processing steps were as follows:

(1) Left–right flipping and data extraction: considering that cranial reconstruction and postoperative scalp conditions may affect light scattering and absorption, thereby impacting signal quality, fNIRS data from participants with left-sided cranioplasty were flipped along the left–right axis using MATLAB (2024a) (31, 32). The number of affected participants is 22. Supplementary Table S2 provides detailed infoemation. Data from the flipped left hemisphere were then extracted for subsequent analysis.
(2) Quality control: in fNIRS studies, the coefficient of variation (CV) is typically used to assess the signal quality (33, 34). The CV was calculated for all channels, and channels with a CV greater than 15% were considered low quality (33). Participants were excluded if more than 20% of their channels were low quality. No participants were excluded from this study on this basis.
(3) Spatial registration: anatomical reference points, optode locations, and channel information of the optode montage in the current study were selected.
(4) Optical density calculation: raw fNIRS data were converted into optical density (OD).
(5) Motion artifact correction: motion artifacts were corrected using the spline interpolation method. The parameters were set as follows: tMotion = 0.5, STDEV thresh = 20, tMASK = 3, and AMPthresh = 5.
(6) Filter: an IIR Butterworth bandpass filter was applied to remove low-frequency drifts and high-frequency physiological noise, including cardiac and respiratory signals, with a passband of 0.01–0.10 Hz (35).
(7) Detrending: linear, quadratic, and cubic trends were removed from the data.
(8) Hemoglobin concentration calculation: changes in changes in oxyhemoglobin concentration (ΔHbOC) levels were derived from the multiwavelength (e.g., 690 nm and 830 nm) OD signals using the modified Beer–Lambert law and known extinction coefficients (36). As oxyhemoglobin (Oxy-Hb) is the most sensitive indicator of cortical hemodynamic changes (37, 38), all the metrics and related statistical analyses in this study were based on relative changes in Oxy-Hb levels.
(9) Block averaging: to obtain more stable concentration responses, 60-s segments were extracted for each task marker, including 15 s of pre-task, 30 s of task, and 15 s of posttask data. Each segment was baseline corrected by subtracting the mean of the corresponding pre-task baseline period. The baseline-corrected segments were then averaged across trials to obtain the block-averaged concentration changes.
(10) Calculation of task-related key metrics: task-related key metrics, including β values, peak amplitude, and initial slope, were calculated on the basis of relative changes in ΔHbOC (39, 40). Previous studies have shown that analyzing fNIRS signals at the region-of-interest (ROI) level provides greater reliability, whereas single-channel signals are more susceptible to noise, motion artifacts, and individual anatomical variability. To enhance signal robustness, this study adopted an ROI-level analysis approach: relative ΔHbOC were first averaged across all channels within each ROI, and task-related key metrics were then calculated on the basis of these ROI-averaged signals for subsequent group-level statistical analyses (41, 42).

**β** **Value:** first, a Generalized Liner Model (GLM) was constructed, and the observed fNIRS signal was regarded as a linear superposition result of the variables and error terms of the hemodynamic response caused by different conditions. The typical hemodynamic response function (HRF) was then convolved with the reference time series of different experimental conditions to obtain the ideal hemodynamic response under each condition. The GLM was subsequently used to estimate the model coefficient (i.e., beta value) for each ROI under different experimental conditions, which represents the intensity of the hemodynamic response corresponding to different experimental conditions (39, 43, 44).

**Peak value:** the maximum Oxy-Hb level was extracted for each ROI during the stimulation period, reflecting the highest hemodynamic response achieved in response to the stimulus.

**Initial slope:** the initial slope was calculated as the ΔHbOC between the first and last data points within a specified time window divided by the window duration. This metric reflects the rate of Oxy-Hb level change immediately following task onset, indicating the speed of early cortical activation. Previous studies have shown that cortical Oxy-Hb levels typically peak approximately 6 s after stimulus onset (39); therefore, in this study, the slope of the fitted line was calculated for the 0–6 s period following stimulus onset.

(11) Visilization: the NirMaster (Wuhan Znion Technology Co., Wuhan, China) was used for 3D visualization.

### 2.5 Statistical analysis

Blinding of statistical analysts: the statisticians were blinded to the group assignments and the specific interventions applied to each group.

(1) Demographic and behavioral data: statistical analyses and plotting were performed using SPSS 26.0 or Origin software. The Shapiro–Wilk test was used to assess the normality of continuous variables. Normally distributed data are presented as means ± SDs, whereas non-normally distributed data are presented as medians (P25, P75). Categorical data are expressed as frequencies (percentages).

(2) fNIRS β value: statistical analyses and plotting were performed using SPSS 26.0 or Origin software. The Shapiro–Wilk test was used to assess data normality. Normally distributed data are presented as means ± SDs, and non-normally distributed data are presented as medians (P25, P75). For normally distributed data, paired-sample *t*-tests were used to compare β values under the two auditory stimulation conditions with those at baseline. For non-normally distributed data, the Wilcoxon signed-rank test was applied. Significance was set at α = 0.05, and *P*-values were obtained using a two-sided permutation test with false discovery rate (FDR) correction. Result visualization was conducted via OriginPro 2025 (OriginLab Corporation, Northampton, MA, USA).

(3) fNIRS peak and initial slope value: statistical analyses and plotting were performed using SPSS 26.0 or Origin software. The Shapiro–Wilk test was used to assess data normality. Normally distributed data are presented as means ± SDs, and non-normally distributed data are presented as medians (P25, P75). For normally distributed data, paired-sample *t*-tests were used to compare metrics between the two auditory stimulation conditions. For non-normally distributed data, the Wilcoxon signed-rank test was applied. Significance was set at α = 0.05, and *P*-values were obtained using a two-sided permutation test with FDR correction. Result visualization was conducted via OriginPro2025 (OriginLab Corporation, Northampton, MA, USA).

(4) Correlation analysis between fNIRS key metrics and CRS-*R* Scores: depending on the normality of the data, either Pearson or Spearman correlation analyses were performed to evaluate the relationships between β values, peak values, and initial slope metrics and the total and subscale scores of the CRS-*R*. Significance was set at α = 0.05, and *P*-values were obtained using a two-sided permutation test.

## 3 Results

### 3.1 Participants

A total of 64 participants with DOC were recruited for this study. Four participants were lost to follow-up because of hospital discharge after providing informed consent, leaving 60 participants who completed the CRS-*R* and fNIRS assessments. Fifteen participants were excluded from the final data analysis because of bilateral cortical lesions, resulting in 45 participants being included in the statistical analysis. The demographic characteristics are summarized in Table 1, and Supplementary Table S2 provides detailed demographic and clinical information for the participants with DOC.

**Table 1:** Baseline characteristics of the study participants.

Total pDOC (n)	Gender (M/F, n/%)	Age [years, M (P25, P75)]	Dominant hand (right, n/%)	Etiology of damage (*n*/%)								Lateralization of brain injury (L/*R*, *n*/%)	Duration of disease [days, M (P25, *P* 75)]	Type of pDOC (*n*/%)			Baseline CRS-*R* (total score, mean ± SD)
				Hemorrhagic	Ischemic	TBI	SAH	Hemorrhagic/ SAH	HIE	Ischemic/ Hemorrhagic	TBI/SAH			Coma	UVS/VS	MCS	
45	33/12 (73.3/26.7)	66 (47.25, 69.75)	45 (100%)	22 (48.9)	8 (17.8)	7 (15.6)	3 (6.7)	2 (4.4)	1 (2.2)	1 (2.2)	1 (2.2)	22/23 (48.9/51.1)	77 (44.75, 144.25)	6 (13.3)	12 (26.7)	27 (60)	8.64 ± 4.74

pDOC, prolonged disorders of consciousness; M, male; F, female; TBI, traumatic brain injury; SAH, subarachnoid hemorrhage; HIE, hypoxic-ischemic encephalopathy; L, left; R, right; VS, vegetative state; UWS, unresponsive wakefulness syndrome; MCS, minimally conscious state; CRS-R, coma recovery scale-revised.

### 3.2 ROI activation

**Figure 3:**
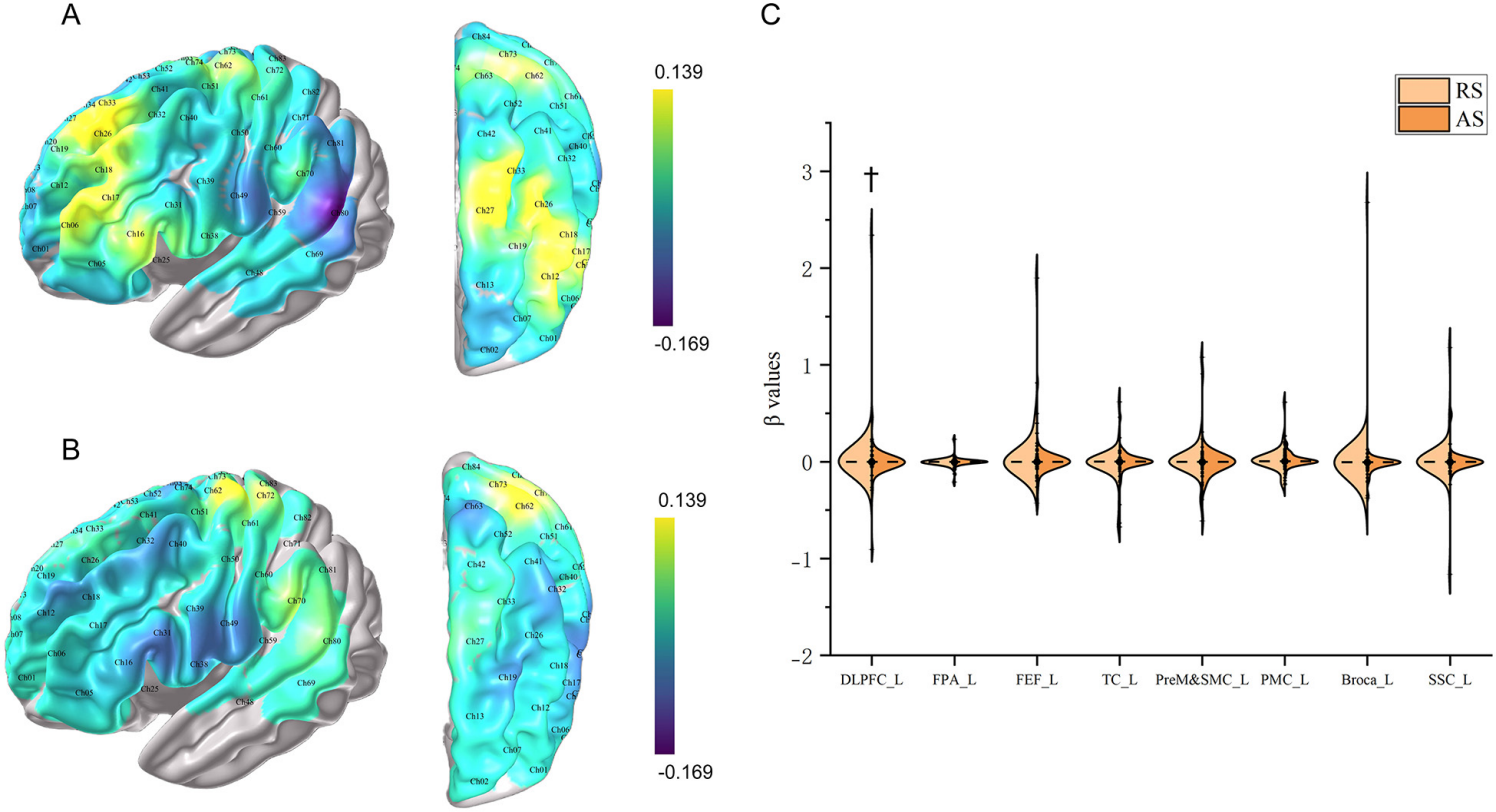
ROI activation of the ROIs under the UFV SON stimulation paradigm. **(A)** ROI activation under the rest state of the UFV SON stimulation paradigm. **(B)** ROI activation under the auditory stimulation of the UFV SON stimulation paradigm. **(C)** Analyze the differences in ROI activation during AS compared to RS under the UFV SON stimulation paradigm; ^†^ denotes after Benjamini–Hochberg correction 0.01 < *P* < 0.05. unfamiliar voice of subject's own name (UFV SON), familiar voice of subject's own name (FV SON), rest state (RS), auditory stimulation (AS), dorsolateral pre-frontal cortex (DLPFC): Ch5, Ch6, Ch12, Ch19, Ch26, Ch27, Ch32, Ch18, frontal pole area (FPA): Ch1, Ch2, Ch7, Ch13, frontal eye field (FEF): Ch33, Ch41, Ch42, temporal cortex (TC): Ch48, Ch69, Ch59, Ch80, pre-motor and supplementary motor cortex (PreM & SMC): Ch38, Ch39, Ch40, Ch50, Ch51, Ch52, Ch62, Ch63, Ch73, Ch49, primary motor cortex (PMC): Ch84, Ch61, Ch72, Ch83, Broca's area: Ch31, Ch16, Ch17, Ch25, somatosensory cortex (SSC): Ch81, Ch82, Ch70, Ch71, Ch60.

**Figure 4:**
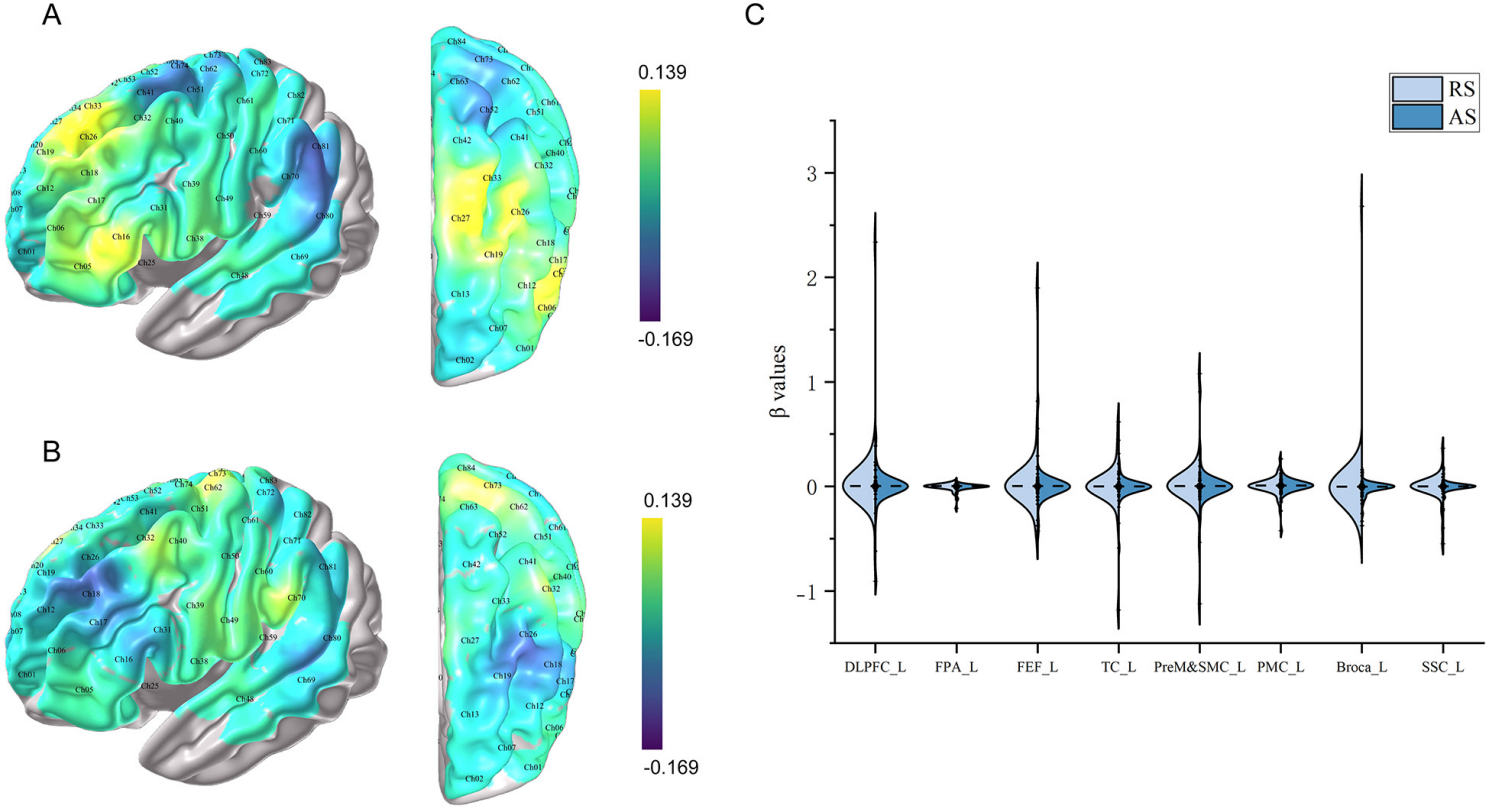
ROI activation of the ROIs under the FV SON stimulation paradigm. **(A)** ROI activation under the rest state of the FV SON stimulation paradigm. **(B)** ROI activation under the auditory stimulation of the FV SON stimulation paradigm. **(C)** Analyze the differences in ROI activation during AS compared to RS under the FV SON stimulation paradigm. unfamiliar voice of subject's own name (UFV SON), familiar voice of subject's own name (FV SON), rest state (RS), auditory stimulation (AS), dorsolateral pre-frontal cortex (DLPFC): Ch5, Ch6, Ch12, Ch19, Ch26, Ch27, Ch32, Ch18, frontal pole area (FPA): Ch1, Ch2, Ch7, Ch13, frontal eye field (FEF): Ch33, Ch41, Ch42, temporal cortex (TC): Ch48, Ch69, Ch59, Ch80, pre-motor and supplementary motor cortex (PreM & SMC): Ch38, Ch39, Ch40, Ch50, Ch51, Ch52, Ch62, Ch63, Ch73, Ch49, primary motor cortex (PMC): Ch84, Ch61, Ch72, Ch83, Broca's area: Ch31, Ch16, Ch17, Ch25, somatosensory cortex (SSC): Ch81, Ch82, Ch70, Ch71, Ch60.

As shown in Table 2 and Figures 3, 4, in the block design, compared with the rest state (RS), participants with DOC under UFV SON stimulation exhibited deactivation in the DLPFC-L (*P* = 0.0048, *P* (FDR) = 0.0381, *Z* = −1.9810, *r* = −0.2953). However, compared with the RS, participants with DOC under FV SON stimulation did not show deactivation in the DLPFC-L (*P* = 0.9057, *P* (FDR) = 0.9685, *Z* = −0.1185, *r* = −0.0177). In addition, compared with the RS, participants with DOC did not show activation or deactivation in the FPA-L, Left Frontal Eye Field (FEF-L), Left Temporal Cortex (TC-L), Left Pre-Motor Cortex and Supplementary Motor Cortex (PreM & SMC-L), Left primary motor cortex (PMC-L), Left Broca's area (Broca-L) or SSC-L, whether under UFV SON stimulation or under FV SON stimulation.

**Table 2:** Results of ROI activation.

ROIs	Stimulation	β values (rest state)	β values (auditory stimulation)	*P*-values	*P*-values (FDR)	*Z*	*r*
DLPFC-L	FV [M (P25, P75)]	0.001085 (−0.016012, 0.017252)	0.003574 (−0.013230, 0.017058)	0.905656^*^	0.9685	−0.118519^**#**^	−0.017668
	95 % CI	−0.050332 to 0.171531	−0.058006 to 0.041400				
	UFV [M (P25, P75)]	−0.001552 (−0.017952, 0.015016)	−0.003712 (−0.010291, 0.004568)	0.004760^*^†	0.0381^**†**^	−0.852210^**##**^	−0.295305
	95 % CI	−0.046771 to 0.168007	−0.066998 to 0.030903				
FPA-L	FV [M (P25, P75)]	0.004104 (−0.008004, 0.010007)	0.001903 (−0.007455, 0.008622)	0.869991^*^	0.9685	−0.163669^**#**^	−0.127040
	95 % CI	−0.016426 to 0.007504	−0.014050 to 0.003532				
	UFV [M (P25, P75)]	−0.002209 (−0.012873, 0.008419)	−0.000217 (−0.011513, 0.010216)	0.896719^*^	0.5337	−0.129807^**#**^	−0.190981
	95 % CI	−0.018824 to 0.012528	−0.012078 to 0.004421				
FEF-L	FV [M (P25, P75)]	0.003180 (−0.018039, 0.023745)	0.001761 (−0.023692, 0.015141)	0.538441^*^	0.9685	−0.615172^**##**^	−0.024398
	95 % CI	−0.048520 to 0.141113	−0.047819 to 0.043725				
	UFV [M (P25, P75)]	−0.001794 (−0.028308, 0.037394)	0.001074 (−0.011283, 0.018475)	0.968487^*^	0.7138	−0.039506^**##**^	−0.113579
	95 % CI	−0.033919 to 0.151953	−0.040422 to 0.050340				
TC–L	FV [M (P25, P75)]	−0.001214 (−0.015782, 0.010605)	0.000750 (−0.013147, 0.010460)	0.286119^*^	0.9685	−1.066674^**#**^	−0.019350
	95 % CI	−0.072622 to 0.065920	−0.040000 to 0.020973				
	UFV [M (P25, P75)]	−0.001086 (−0.016650, 0.016935)	0.003845 (−0.008142, 0.017970)	0.950498^*^	0.9057	−0.062082^**##**^	−0.058051
	95 % CI	−0.084348 to 0.028786	−0.014177 to 0.039295				
PreM & SMC–L	FV [M (P25, P75)]	0.005018 (−0.020754, 0.039305)	0.001036 (−0.010762, 0.013180)	0.905656^*^	0.9685	−0.118519^**#**^	−0.091704
	95 % CI	−0.078536 to 0.075599	−0.025477 to 0.069326				
	UFV [M (P25, P75)]	−0.001235 (−0.029140, 0.046954)	−0.001223 (−0.019264, 0.008236)	0.394097^*^	0.5337	−0.852210^**##**^	−0.209490
	95 % CI	−0.035795 to 0.082534	−0.062759 to 0.046475				
PMC–L	FV [M (P25, P75)]	0.005504 (−0.014718, 0.041172)	0.008611 (−0.007997, 0.032012)	0.869991^*^	0.9685	−0.163669^**#**^	−0.005889
	95 % CI	−0.016182 to 0.031982	−0.019692 to 0.025925				
	UFV [M (P25, P75)]	0.005795 (−0.008126, 0.057776)	0.006622 (−0.011649, 0.040776)	0.896719^*^	0.9057	−0.129807^**#**^	−0.017668
	95 % CI	−0.016323 to 0.060885	0.001558 to 0.036230				
Broca–L	FV [M (P25, P75)]	−0.002361 (−0.019179, 0.007054)	−0.000832 (−0.024319, 0.009027)	0.538441^*^	0.9685	−0.615172^**##**^	−0.159010
	95 % CI	−0.087881 to 0.159755	−0.034807 to 0.001534				
	UFV [M (P25, P75)]	−0.005035 (−0.017301, 0.008256)	−0.004795 (−0.035834, 0.002519)	0.968487^*^	0.7138	−0.039506^**##**^	−0.123675
	95 % CI	−0.087715 to 0.160140	−0.047353 to −0.004549				
SSC–L	FV [M (P25, P75)]	−0.000260 (−0.015845, 0.023967)	0.001989 (−0.010481, 0.012356)	0.286119^*^	0.9685	−1.066674^**#**^	−0.009255
	95 % CI	−0.051604 to 0.027457	−0.009578 to 0.015476				
	UFV [M (P25, P75)]	−0.002099 (−0.020548, 0.014018)	0.000734 (−0.015615, 0.011331)	0.950498^*^	0.9057	−0.062082^**##**^	−0.019350
	95 % CI	−0.069127 to 0.090205	−0.018573 to 0.045940				

^*^Wilcoxon signed–rank test.

^**#**^denotes negative rank.

^**##**^denotes positive rank.

^**‡**^denotes after Benjamini–Hochberg correction 0.01 < P < 0.05.

ROIs, reigns of interest; DLPFC–L, left dorsolateral pre–frontal cortex; FPA-L, left frontal pole area; FEF-L, left frontal eye field; TC-L, left temporal cortex; PreM & SMC-L, left pre-motor cortex and supplementary motor cortex; PMC-L, left primary motor cortex; Broca-L, left Broca's area; SSC-L, left somatosensory cortex.

### 3.3 Peak values of the ROIs

**Figure 5:**
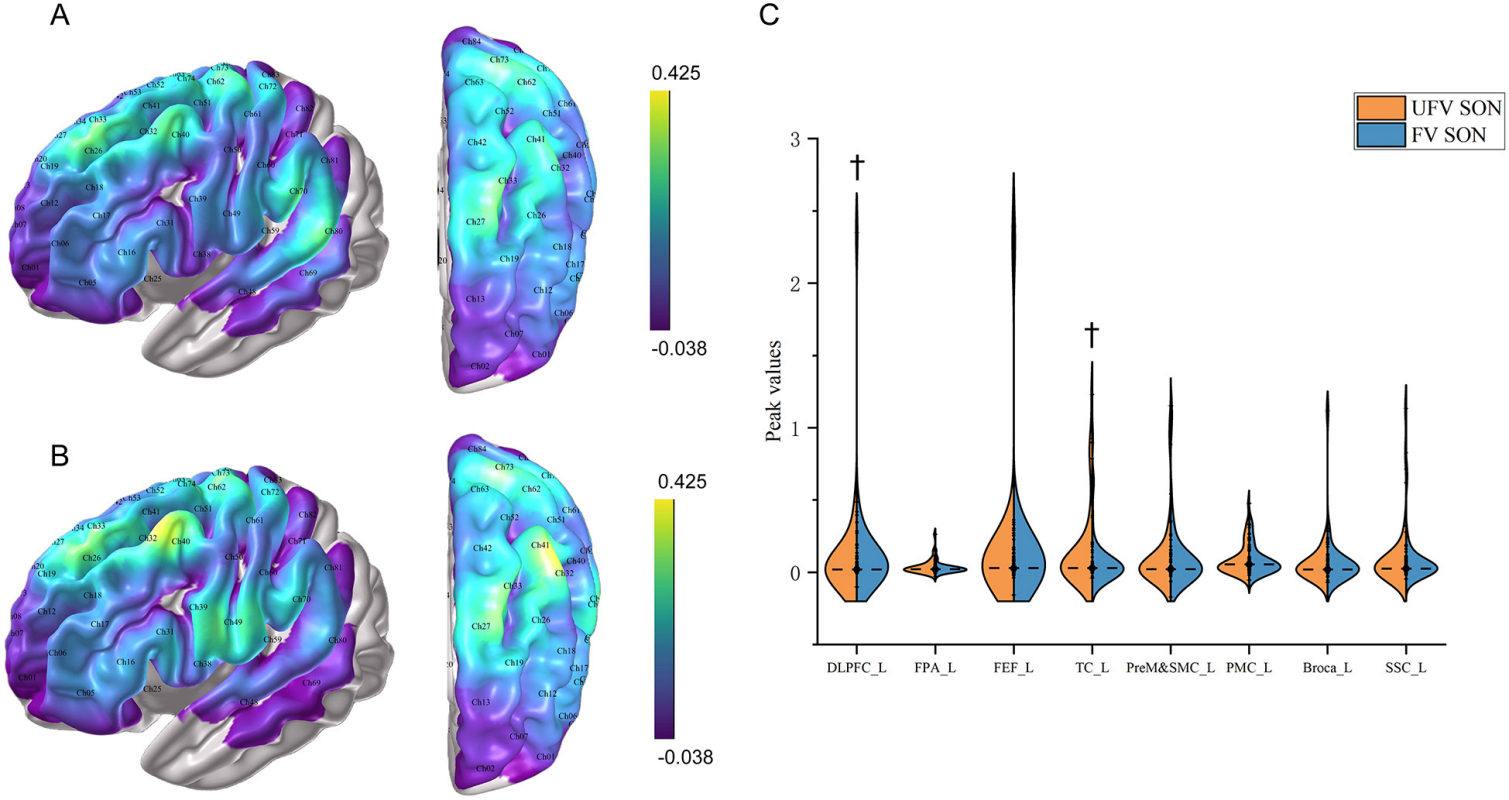
Peak values of the ROIs. **(A)** Peak values of the ROIs under the auditory stimulation of the UFV SON stimulation paradigm. **(B)** Peak values of the ROIs under the auditory stimulation of the FV SON stimulation paradigm. **(C)** Analyze the differences in peak values during AS under the UFV SON stimulation paradigm compared to the FV SON stimulation paradigm; ^†^ denotes after Benjamini-Hochberg correction 0.01 < *P* < 0.05. unfamiliar voice of subject's own name (UFV SON), familiar voice of subject's own name (FV SON), rest state (RS), auditory stimulation (AS), dorsolateral pre-frontal cortex (DLPFC): Ch5, Ch6, Ch12, Ch19, Ch26, Ch27, Ch32, Ch18, frontal pole area (FPA): Ch1, Ch2, Ch7, Ch13, frontal eye field (FEF): Ch33, Ch41, Ch42, temporal cortex (TC): Ch48, Ch69, Ch59, Ch80, pre-motor and supplementary motor cortex (PreM & SMC): Ch38, Ch39, Ch40, Ch50, Ch51, Ch52, Ch62, Ch63, Ch73, Ch49, primary motor cortex (PMC): Ch84, Ch61, Ch72, Ch83, Broca's area: Ch31, Ch16, Ch17, Ch25, somatosensory cortex (SSC): Ch81, Ch82, Ch70, Ch71, Ch60.

As shown in Table 3 and Figure 5, with respect to the block design, compared with the UFV SON stimulation, the peak value of participants with DOC under FV SON stimulation was greater in the DLPFC-L (*P* = 0.0016, *P* (FDR) = 0.0126, *Z* = −2.4146, *r* = −0.3485). However, compared with the FV SON stimulation, the peak value of participants with DOC under UFV SON stimulation was greater for the TC-L (*P* = 0.0049, *P* (FDR) = 0.0198, *Z* = −1.9650, *r* = −0.2836). In addition, there was no significant difference in the peak values of the participants with DOC under FV SON stimulation or UFV SON stimulation in the FPA-L, FEF-L, PreM & SMC-L, PMC-L, Broca-L or SSC-L.

**Table 3:** Results of peak values.

ROIs	Stimulation	Peak values (auditory stimulation)	*P*-values	*P*-values (FDR)	*Z*	*r*
DLPFC-L	FV [M (P25, P75)]	0.029401 (0.012351, 0.071956)	0.001575^*^	0.0126^†^	−2.414629^**#**^	−0.359952
	95 % CI	0.011759 to 0.224746				
	UFV [M (P25, P75)]	0.015413 (0.003608, 0.041361)				
	95 % CI	−0.003429 to 0.213396				
FPA-L	FV [M (P25, P75)]	0.021197 (0.009732, 0.038188)	0.412570^*^	0.8251	−0.819379^**##**^	−0.122146
	95 % CI	0.018460 to 0.037975				
	UFV [M (P25, P75)]	0.023945 (0.009904, 0.043428)				
	95 % CI	0.023804 to 0.053836				
FEF-L	FV [M (P25, P75)]	0.033899 (0.006647, 0.098514)	0.722361^*^	0.9595	−0.355306^**#**^	−0.052966
	95 % CI	0.026808 to 0.311314				
	UFV [M (P25, P75)]	0.027690 (0.006071, 0.092355)				
	95 % CI	0.014308 to 0.299488				
TC-L	FV [M (P25, P75)]	0.023641 (0.007069, 0.060117)	0.004941^*^	0.0198^†^	−1.965058^**##**^	−0.292934
	95 % CI	0.031482 to 0.145488				
	UFV [M (P25, P75)]	0.033654 (0.011021, 0.165590)				
	95 % CI	0.068781 to 0.241003				
PreM & SMC-L	FV [M (P25, P75)]	0.023118 (0.007591, 0.080150)	0.627090^*^	0.9595	−0.485826^**#**^	−0.072423
	95 % CI	0.037013 to 0.182901				
	UFV [M (P25, P75)]	0.020235 (0.005609, 0.088917)				
	95 % CI	0.022415 to 0.148384				
PMC-L	FV [M (P25, P75)]	0.058314 (0.015866, 0.101848)	0.867546^*^	0.9595	−0.166776^**#**^	−0.024862
	95 % CI	0.052641 to 0.110989				
	UFV [M (P25, P75)]	0.040332 (0.017754, 0.104996)				
	95 % CI	0.054192 to 0.118866				
Broca-L	FV [M (P25, P75)]	0.023350 (0.004757, 0.054151)	0.273551^*^	0.7295	−1.094922^**#**^	−0.163221
	95 % CI	0.014440 to 0.120051				
	UFV [M (P25, P75)]	0.015358 (0.005352, 0.068295)				
	95 % CI	0.013295 to 0.117574				
SSC-L	FV [M (P25, P75)]	0.029330 (0.015250, 0.063790)	0.959518^*^	0.9595	−0.050758^**##**^	−0.007567
	95 % CI	0.030689 to 0.115977				
	UFV [M (P25, P75)]	0.018503 (0.003484, 0.089724)				
	95 % CI	0.027299 to 0.153295				

^*^Wilcoxon signed-rank test.

^**#**^denotes negative rank.

^**##**^denotes positive rank.

^†^denotes after Benjamini-Hochberg correction 0.01 < P < 0.05

ROIs, reigns of Interest; DLPFC-L, left dorsolateral pre-frontal cortex; FPA-L, left frontal pole area; FEF-L, left frontal eye field; TC-L, left temporal cortex; PreM & SMC-L, left pre-motor cortex and supplementary motor cortex; PMC-L, left primary motor cortex; Broca-L, left Broca's area; SSC-L, left somatosensory cortex.

### 3.4 Initial slope of the ROIs

**Figure 6:**
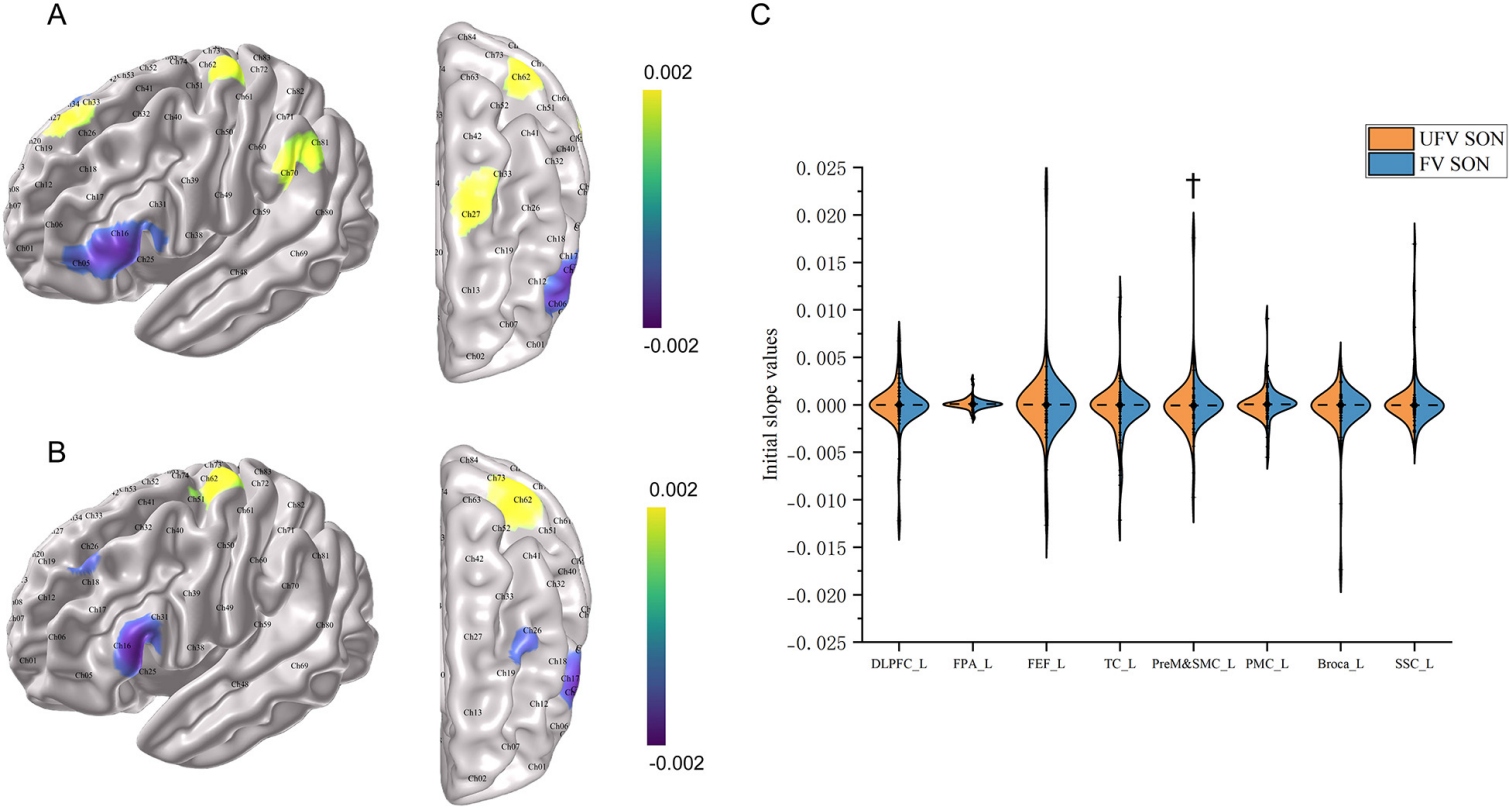
Initial slope values of the ROIs. **(A)** Initial slope values of the ROIs under the auditory stimulation of the UFV SON stimulation paradigm. **(B)** Initial slope values of the ROIs under the auditory stimulation of the FV SON stimulation paradigm. **(C)** Analyze the differences in initial slope values during AS under the UFV SON stimulation paradigm compared to the FV SON stimulation paradigm; ^†^ denotes after Benjamini-Hochberg correction 0.01 < *P* < 0.05. unfamiliar voice of subject's own name (UFV SON), familiar voice of subject's own name (FV SON), rest state (RS), auditory stimulation (AS), dorsolateral pre-frontal cortex (DLPFC): Ch5, Ch6, Ch12, Ch19, Ch26, Ch27, Ch32, Ch18, frontal pole area (FPA): Ch1, Ch2, Ch7, Ch13, frontal eye field (FEF): Ch33, Ch41, Ch42, temporal cortex (TC): Ch48, Ch69, Ch59, Ch80, pre-motor and supplementary motor cortex (PreM & SMC): Ch38, Ch39, Ch40, Ch50, Ch51, Ch52, Ch62, Ch63, Ch73, Ch49, primary motor cortex (PMC): Ch84, Ch61, Ch72, Ch83, Broca's area: Ch31, Ch16, Ch17, Ch25, somatosensory cortex (SSC): Ch81, Ch82, Ch70, Ch71, Ch60.

As shown in Table 4 and Figure 6, with respect to the block design, compared with the UFV SON stimulation, the initial slope of participants with DOC under FV SON stimulation was greater for PreM & SMC-L (*P* = 0.0022, *P* (FDR) = 0.0172, *Z* = −2.2986, *r* = −0.3427). However, there was no significant difference in the initial slope of the participants with DOC under FV SON stimulation or UFV SON stimulation in the DLPFC-L, FPA-L, FEF-L, TC-L, PMC-L, Broca-L or SSC-L.

**Table 4:** Results of initial slope.

ROIs	Stimulation	Initial slope (auditory stimulation)	*P*-values	*P*-values (FDR)	*Z*	*r*
DLPFC-L	FV [M (P25, P75)]	0.000004 (−0.000426, 0.000163)	0.586555^*^	0.6067	−0.543835^**#**^	−0.081070
	95 % CI	−0.000900 to 0.000633				
	UFV [M (P25, P75)]	−0.000009 (−0.000181, 0.000212)				
	95 % CI	−0.000907 to 0.000661				
FPA-L	FV [M (P25, P75)]	0.000059 (−0.000155, 0.000211)	0.518700^*^	0.6067	−0.645351^**#**^	−0.096203
	95 % CI	−0.000108 to 0.000205				
	UFV [M (P25, P75)]	0.000074 (−0.000052, 0.000277)				
	95 % CI	−0.000016 to 0.000314				
FEF-L	FV [M (P25, P75)]	0.000024 (−0.000472, 0.000389)	0.372452^*^	0.6067	−0.891890^**##**^	−0.132955
	95 % CI	−0.001282 to 0.001342				
	UFV [M (P25, P75)]	−0.000030 (−0.000227, 0.000438)				
	95 % CI	−0.000992 to 0.001497				
TC-L	FV [M (P25, P75)]	−0.000033 (−0.000403, 0.000070)	0.334845^*^	0.6067	−0.964401^**#**^	−0.143764
	95 % CI	−0.001405 to 0.000279				
	UFV [M (P25, P75)]	0.000017 (−0.000302, 0.000175)				
	95 % CI	−0.001073 to 0.000540				
PreM & SMC-L	FV [M (P25, P75)]	0.000017 (−0.000138, 0.000431)	0.002153^*^	0.0172^†^	−2.298611^**##**^	−0.342657
	95 % CI	−0.000385 to 0.001428				
	UFV [M (P25, P75)]	−0.000094 (−0.000499, 0.000125)				
	95 % CI	−0.001103 to 0.000929				
PMC-L	FV [M (P25, P75)]	0.000134 (−0.000150, 0.000457)	0.566753^*^	0.6067	−0.572840^**##**^	−0.085394
	95 % CI	−0.000562 to 0.000322				
	UFV [M (P25, P75)]	−0.000056 (−0.000488, 0.000322)				
	95 % CI	−0.000213 to 0.000819				
Broca-L	FV [M (P25, P75)]	−0.000078 (−0.000382, 0.000124)	0.279955^*^	0.6067	−1.080420^**#**^	−0.161059
	95 % CI	−0.001733 to 0.000129				
	UFV [M (P25, P75)]	0.000003 (−0.000325, 0.000149)				
	95 % CI	−0.001276 to 0.000455				
SSC-L	FV [M (P25, P75)]	−0.000030 (−0.000234, 0.000097)	0.606671^*^	0.6067	−0.514831^**#**^	−0.076746
	95 % CI	−0.000568 to 0.001128				
	UFV [M (P25, P75)]	−0.000057 (−0.000348, 0.000197)				
	95 % CI	−0.000478 to 0.000964				

^*^Wilcoxon signed-rank test

^**#**^denotes negative rank

^**##**^denotes positive rank

^**‡**^denotes after Benjamini-Hochberg correction 0.01 < P < 0.05

ROIs, reigns of Interest; DLPFC-L, left dorsolateral pre-frontal cortex; FPA-L, left frontal pole area; FEF-L, left frontal eye field; TC-L, left temporal cortex; PreM & SMC-L, left pre-motor cortex and supplementary motor cortex; PMC-L, left primary motor cortex; Broca-L, left Broca's area; SSC-L, left somatosensory cortex.

### 3.5 Correlations between peak values of the ROIs and CRS-*R* scores

The correlations between peak values and CRS-*R* scores for participants under FV and UFV SON stimulation are presented in Table 5.

**Table 5:** Correlation between peak values and CRS-*R* Scores.

ROIs	Stimulation	Statistical indices	CRS-*R*						
			Total	AFC	VFS	MFS	OFS	CS	AS
DLPFC-L	FV	Correlation coefficient	0.271587	0.259772	0.315201	0.235717	−0.030387	−0.265219	0.288300
		*P*	0.071124	0.084837	0.034942^*^	0.119044	0.842927	0.078279	0.054789
		95 % CI	−0.032781 to 0.529857	−0.045475 to 0.520652	0.014939 to 0.563318	−0.071015 to I60.501722	−0.329053 to 0.273806	−0.524903 to 0.039635	−0.014658 to 0.542774
	UFV	Correlation coefficient	0.151396	0.118872	0.229023	0.068099	−0.034578	−0.012833	0.156051
		*P*	0.320830	0.436723	0.130206	0.656692	0.821592	0.933320	0.306000
		95 % CI	−0.157479 to 0.433289	−0.189611 to 0.405995	−0.078053 to 0.496407	−0.238483 to 0.362338	−0.332789 to 0.269920	−0.313303 to 0.289972	−0.152827 to 0.437153
FPA-L	FV	Correlation coefficient	0.078210	0.062068	0.123933	0.058468	−0.015717	−0.226719	0.041979
		*P*	0.609582	0.685448	0.417305	0.702827	0.918380	0.134221	0.784236
		95 % CI	−0.228873 to 0.371136	−0.244187 to 0.357065	−0.184654 to 0.410276	−0.247581 to 0.353908	−0.315902 to 0.287328	−0.494575 to 0.080467	−0.263034 to 0.339364
	UFV	Correlation coefficient	0.211227	0.136328	0.176005	0.263859	0.000000	−0.012833	0.186095
		*P*	0.163664	0.371878	0.247473	0.079878	1.000000	0.933320	0.220968
		95 % CI	−0.096612 to 0.482183	−0.172447 to 0.420708	−0.132725 to 0.453602	−0.041095 to 0.523843	−0.301683 to 0.301683	−0.313303 to 0.289972	−0.122463 to 0.461849
FEF–L	FV	Correlation coefficient	0.104192	0.084789	0.107859	0.179162	−0.080682	−0.299441	0.096031
		*P*	0.495789	0.579731	0.480661	0.238960	0.598288	0.045684^*^	0.530317
		95 % CI	−0.203900 to 0.393506	−0.222587 to 0.376833	−0.200343 to 0.396635	−0.129522 to 0.456187	−0.373279 to 0.226514	−0.551319 to 0.002465	−0.211787 to 0.386516
	UFV	Correlation coefficient	0.202236	0.257416	0.261846	0.191661	−0.207469	−0.149720	0.203792
		*P*	0.182769	0.087802	0.082290	0.207217	0.171464	0.326277	0.179353
		95 % CI	−0.105908 to 0.474942	−0.047993 to 0.518809	−0.043253 to 0.522272	−0.116773 to 0.466378	−0.479161 to 0.100504	−0.431895 to 0.159151	−0.104303 to 0.476198
TC–L	FV	Correlation coefficient	0.274430	0.252290	0.366733	0.148835	−0.114213	−0.076999	0.335835
		*P*	0.068102	0.094533	0.013216	0.329180	0.455032	0.615149	0.024108^*^
		95 % CI	−00.029713 to 00.532063	−0.053461 to 00.514791	0.073143 to 0.601831	−0.160034 to 0.431158	−0.402043 to 0.194160	−0.370085 to 0.230027	0.038003 to 0.578869
	UFV	Correlation coefficient	0.341599	0.263651	0.429544	0.217139	−0.005239	−0.021389	0.358883
		*P*	0.021642^*^	0.080124	0.003234^**^	0.151921	0.972753	0.889089	0.015479^*^
		95 % CI	0.044503 to 0.583182	−0.041318 to 0.523681	0.146897 to 0.647340	−0.090470 to 0.486924	−0.306437 to 0.296913	−0.321000 to 0.282114	0.064145 to 0.596033
PreM & SMC–L	FV	Correlation coefficient	−0.026907	−0.092409	0.000608	−0.008470	−0.032483	−0.166831	0.016943
		*P*	0.860724	0.546009	0.996838	0.955964	0.832245	0.273366	0.912041
		95 % CI	−0.325944 to 0.277024	−0.383403 to 0.215275	−0.301130 to 0.302235	−0.309362 to 0.293964	−0.330922 to 0.271864	−0.446064 to 0.141998	−0.286203 to 0.317005
	UFV	Correlation coefficient	0.157346	0.135635	0.152907	0.066596	0.023052	0.141165	0.181911
		*P*	0.301953	0.374336	0.315968	0.663813	0.880523	0.354987	0.231711
		95 % CI	−0.151530 to 0.438227	−0.173132 to 0.420127	−0.155971 to 0.434544	−0.239906 to 0.361026	−0.280582 to 0.322492	−0.167658 to 0.424758	−0.126727 to 0.458435
PMC–L	FV	Correlation coefficient	−0.235622	−0.201860	−0.113464	−0.132442	−0.339495	−0.436328	−0.189730
		*P*	0.119197	0.183601	0.458011	0.385792	0.022516^*^	0.002732^**^	0.211918
		95 % CI	−0.501646 to 0.071115	−0.474638 to 0.106296	−0.401407 to 0.194889	−0.417445 to 0.176285	−0.581609 to −0.042127	−0.652165 to −0.155056	−0.464808 to 0.118749
	UFV	Correlation coefficient	0.300676	0.329598	0.364504	0.230048	−0.139361	−0.012833	0.278765
		*P*	0.044755^*^	0.027037^*^	0.013828^*^	0.128447	0.361233	0.933320	0.063691
		95 % CI	−0.001107 to 0.552264	0.030998 to 0.574187	0.070583 to 0.600188	−0.076977 to 0.497223	−0.423249 to 0.169446	−0.313303 to 0.289972	−0.025022 to 0.535420
Broca–L	FV	Correlation coefficient	−0.036824	−0.066917	−0.063081	−0.067963	−0.086969	0.047055	0.048290
		*P*	0.810210	0.662291	0.680587	0.657338	0.569988	0.758888	0.752756
		95 % CI	−0.334788 to 0.267834	−0.361306 to 0.239603	−0.357952 to 0.243231	−0.362219 to 0.238612	−0.378716 0.220499	−0.258294 to 0.343856	−0.257139 to 0.344947
	UFV	Correlation coefficient	0.346095	0.258109	0.350119	0.271850	0.073348	0.042777	0.373493
		*P*	0.019868^*^	0.086921	0.018386^*^	0.070840	0.632059	0.780236	0.011499^*^
		95 % CI	0.049590 to 0.586537	−0.047253 to 0.519351	0.054156 to 0.589532	−0.032498 to 0.530061	–.233502 to 0.366911	−0.262290 to 0.340071	0.080929 to 0.606803
SSC–L	FV	Correlation coefficient	0.127463	0.089777	0.186946	0.083126	−0.192800	−0.145443	0.204066
		*P*	0.404055	0.557552	0.218825	0.587215	0.204481	0.340445	0.178756
		95 % CI	−0.181186 to 0.413255	−0.217805 to 0.381137	−0.121594 to 0.462542	−0.224178 to 0.375395	−0.467302 to 0.115607	−0.428331 to 0.163410	−0.104020 to 0.476419
	UFV	Correlation coefficient	0.243820	0.227075	0.265966	0.207986	−0.078587	0.076999	0.213121
		*P*	0.106520	0.133595	0.077411	0.170376	0.607856	0.615149	0.159832
		95 % CI	−0.062457 to 0.508127	−0.080094 to 0.494858	−0.038832 to 0.525485	−0.099969 0.479577	−0.371463 to 0.228514	−0.230027 to 0.370085	−0.094647 to 0.483704

^*^Correlation coefficient is represented by the Spearman's rank correlation coefficient.

^*^denotes 00.01 < P < 00.05.

^**^denotes 00.001 < P < 00.010.

ROIs, reigns of interest; DLPFC-L, left dorsolateral pre-frontal cortex; FPA-L, left frontal pole area; FEF-L, left frontal eye field; TC-L, left temporal cortex; PreM & SMC-L, left pre-motor cortex and supplementary motor cortex; PMC-L, left primary motor cortex; Broca-L, left Broca's area; SSC-L, left somatosensory cortex.

Under FV SON stimulation, the peak values of the DLPFC-L and TC-L were positively correlated with the CRS-*R* VFS score (*P* < 0.05), and the peak value of the PMC-L was negatively correlated with the CRS-*R* OFS score and CRS-*R* CS score (*P* < 0.05).

Under UFV SON stimulation, the peak value of the TC-L was positively correlated with the CRS-*R* total score, CRS-*R* VFS score and CRS-*R* AS score (*P* < 0.05); the peak value of the PMC-L was positively correlated with the CRS-*R* total score, CRS-*R* AFC score and CRS-*R* VFS score (*P* < 0.05); and the peak value of the Broca-L was positively correlated with the CRS-*R* total score, CRS-*R* VFS score and CRS-*R* AS score (*P* < 0.05).

No significant correlations were observed for the remaining indicators.

### 3.6 Correlations between the initial slope of the ROIs and CRS-*R* scores

Table 6 presents the correlations between the initial slope and CRS-*R* scores for participants under FV and UFV SON stimulation.

**Table 6:** Correlation between initial slope and CRS-*R* Scores.

ROIs	Stimulation	Statistical indices	CRS-*R*						
			Total	AFC	VFS	MFS	OFS	CS	AS
DLPFC-L	FV	Correlation coefficient	−0.210896	−0.050430	−0.178706	−0.183123	−0.111069	−0.098388	−0.286516
		*P*	0.164340	0.742166	0.240175	0.228561	0.467617	0.520227	0.056373
		95 % CI	−0.481918 to 0.096955	−0.346836 to 0.255134	−0.455815 to 0.129984	−0.459425 to 0.125492	−0.399370 to 0.197222	−0.399370 to 0.197222	−0.541402 to 0.016602
	UFV	Correlation coefficient	0.225111	0.314358	0.163578	0.202385	0.071252	0.102665	0.233562
		*P*	0.137081	0.035459^*^	0.282963	0.182440	0.641849	0.502159	0.122556
		95 % CI	−0.082151 to 0.493293	0.014004 to 0.562679	−0.145274 to 0.443380	−0.105755 to 0.475062	−0.235493 to 0.365087	−0.205378 to 0.392201	−0.073285 to 0.500013
FPA–L	FV	Correlation coefficient	−0.024594	0.122335	0.015669	−0.020150	−0.279769	−0.316552	−0.005145
		*P*	0.872597	0.423381	0.918631	0.895476	0.062702	0.034128^*^	0.973244
		95 % CI	−0.323873 to 0.279160	−0.186220 to 0.408926	−0.287372 to 0.315858	−0.319888 to 0.283255	−0.536196 to 0.023934	−0.564341 to −0.016439	−0.306352 to 0.296999
	UFV	Correlation coefficient	0.218235	0.186343	0.193092	0.195418	0.150887	0.076999	0.180059
		*P*	0.149813	0.220342	0.203782	0.198283	0.322480	0.615149	0.236578
		95 % CI	−0.089329 to 0.487802	−0.122210 to 0.462051	−0.115307 to 0.467539	−0.112922 to 0.469426	−0.157988 to 0.432865	−0.230027 to 0.370085	−0.128610 to 0.456921
FEF–L	FV	Correlation coefficient	0.063467	0.150460	0.038632	0.017554	0.097448	0.098388	−0.004870
		*P*	0.678736	0.323867	0.801078	0.908880	0.524241	0.520227	0.974671
		95 % CI	−0.242866 to 0.358290	−0.158413 to 0.432510	−0.266153 to 0.336394	−0.285641 to 0.317555	−0.210421 to 0.387732	−0.209514 to 0.388538	−0.306103 to 0.297249
	UFV	Correlation coefficient	0.105052	0.125383	0.137373	0.166662	−0.028291	0.034222	0.029564
		*P*	0.492221	0.411833	0.368189	0.273860	0.853637	0.823402	0.847130
		95 % CI	−0.203067 to 0.394240	−0.183230 to 0.411500	−0.171414 to 0.421584	−0.142169 to 0.445924	−0.327181 to 0.275745	−0.270251 to 0.332472	−0.274568 to 0.328318
TC–L	FV	Correlation coefficient	−0.301205	−0.192993	−0.246920	−0.289200	−0.155078	0.102665	−0.278971
		*P*	0.044362^*^	0.204018	0.102005	0.054003	0.309063	0.502159	0.063487
		95 % CI	−0.552668 to 0.000525	−0.467459 to 0.115409	−0.510570 to 0.059170	−0.543467 to 0.013676	−0.436346 to 0.153800	−0.205378 to 0.392201	−0.535579 to 0.024799
	UFV	Correlation coefficient	−0.016660	0.034636	0.028501	0.001229	−0.158221	0.188220	−0.030387
		*P*	0.913502	0.821298	0.852563	0.993605	0.299237	0.215646	0.842926
		95 % CI	−0.316751 to 0.286462	−0.269866 to 0.332841	−0.275551 to 0.327369	−0.300565 to 0.302800	−0.438952 to 0.150652	−0.120293 to 0.463579	−0.329053 to 0.273806
PreM & SMC–L	FV	Correlation coefficient	0.027569	−0.009837	−0.082194	0.050750	0.223187	−0.068444	0.022293
		*P*	0.857336	0.948866	0.591427	0.740588	0.140559	0.655064	0.884430
		95 % CI	−0.276413 to 0.326535	−0.310598 to 0.292715	−0.374588 to 0.225069	−0.254834 to 0.347118	−0.084163 to 0.491758	−0.362638 to 0.238157	−0.281281 to 0.321811
	UFV	Correlation coefficient	0.008925	0.040871	0.070375	−0.106964	0.198039	0.102665	−0.048221
		*P*	0.953599	0.789804	0.645965	0.484329	0.192213	0.502159	0.753096
		95 % CI	−0.293548 to 0.309774	−0.264068 to 0.338381	−0.236325 to 0.364323	−0.395872 to 0.201211	−0.110230 to 0.471549	−0.205378 to 0.392201	−0.344887 to 0.257203
PMC–L	FV	Correlation coefficient	−0.253274	−0.155170	−0.242598	−0.189954	−0.308060	−0.269497	−0.135473
		*P*	0.093211	0.308771	0.108342	0.211370	0.039519^*^	0.073413	0.374914
		95 % CI	−0.515563 to 0.052413	−0.436423 to 0.153708	−0.507162 to 0.063751	−0.464990 to 0.118521	−0.557894 to −0.007031	−0.528233 to 0.035034	−0.419990 to 0.173292
	UFV	Correlation coefficient	0.095862	0.079248	0.196131	0.038319	−0.074396	0.059888	0.138148
		*P*	0.531045	0.604832	0.196617	0.802659	0.627186	0.695954	0.365468
		95 % CI	−0.211950 to 0.386371	−0.227883 to 0.372036	−0.112189 to 0.470004	−0.266444 to 0.336116	−0.367823 to 0.232505	−0.246243 to 0.355154	−0.170647 to 0.422233
Broca–L	FV	Correlation coefficient	−0.198864	−0.095735	−0.227402	−0.139750	−0.210613	0.012833	−0.160578
		*P*	0.190329	0.531594	0.133022	0.359879	0.164921	0.933320	0.292004
		95 % CI	−0.472217 to 0.109381	−0.386261 to 0.212073	−0.495118 to 0.079752	−0.423575 to 0.169060	−0.481690 to 0.097249	−0.289972 to 0.313303	−0.440902 to 0.148288
	UFV	Correlation coefficient	−0.128852	−0.169856	−0.048290	−0.237630	−0.047152	0.042777	−0.058853
		*P*	0.398912	0.264639	0.752757	0.115992	0.758405	0.780236	0.700960
		95 % CI	−0.414425 to 0.179821	−0.448554 to 0.138947	−0.344947 to 0.257139	−0.503236 to 0.068999	−0.343942 to 0.258204	−0.262290 to 0.340071	−0.354246 to 0.247219
SSC–L	FV	Correlation coefficient	0.007669	0.071489	0.009861	−0.142073	−0.017813	0.038500	0.084782
		*P*	0.960124	0.640738	0.948742	0.351871	0.907542	0.801746	0.579765
		95 % CI	−0.294696 to 0.308638	−0.235268 to 0.365294	−0.292693 to 0.310619	−0.425517 to 0.166758	−0.317788 to 0.285403	−0.266276 to 0.336276	−0.222594 to 0.376826
	UFV	Correlation coefficient	0.137843	0.071351	0.158310	−0.001161	−0.083826	0.243830	0.231984
		*P*	0.366537	0.641387	0.298963	0.993960	0.584061	0.106505	0.125175
		95 % CI	−0.170949 to 0.421978	−0.235399 to 0.365173	−0.150564 to 0.439025	−0.302738 to 0.300627	−0.376000 to 0.223509	−0.062446 to 0.508135	−0.074944 to 0.498761

^*^Correlation coefficient is represented by the Spearman's rank correlation coefficient;

^*^denotes 0.01 < P < 0.05;

^**^denotes 0.001 < P < 0.01.

ROIs, reigns of interest; DLPFC-L, left dorsolateral pre-frontal cortex; FPA-L, left frontal pole area; FEF-L, left frontal eye field; TC-L, left temporal cortex; PreM & SMC-L, left pre-motor cortex and supplementary motor cortex; PMC-L, left primary motor cortex; Broca-L, left Broca's area; SSC-L, left somatosensory cortex.

Under FV SON stimulation, the initial slope of the TC-L was negatively correlated with the CRS-*R* total score (*P* < 0.05), and the peak value of the PMC-L was negatively correlated with the CRS-*R* OFS score (*P* < 0.05).

Under UFV SON stimulation, the initial slope of the DLPFC-L was positively correlated with the CRS-*R* AFC score (*P* < 0.05).

No significant correlations were observed for the remaining indicators.

## 4 Discussion

In this study, fNIRS measurements were conducted on participants with DOC, aiming to systematically investigate the changes in and underlying mechanisms of brain activation patterns during different SON calling stimulation paradigms. Moreover, the correlation between the severity of consciousness impairment and changes in cortical hemodynamic indicators was quantitatively evaluated. The goal is to provide new research perspectives and theoretical foundations for precise assessment, objective quantification, exploration of central mechanisms, and optimization of rehabilitation strategies in DOC.

In participants with DOC, the DLPFC, a core node of the ECN, is responsible for working memory, decision-making, and attentional regulation (45). The present findings revealed that, compared with baseline, the DLPFC-L exhibited deactivation (i.e., decreased Oxy-Hb levels) under UFV SON calling stimulation, whereas this phenomenon was not observed UFV SON calling stimulation. This difference may originate from the dynamic regulatory mechanism of the DLPFC in terms of cognitive resource allocation. Under impaired consciousness, patients' responsiveness to external stimuli is compromised; unfamiliar voices may be perceived as low salience or irrelevant stimuli, thereby triggering an inhibitory DLPFC response (similar to task-negative activation) to reduce cognitive resource consumption. Conversely, familiar voices (e.g., calls from relatives) may activate autobiographical information associated with long-term memory, recruiting the DLPFC for recognition and processing and thus maintaining its activation level (23–26). Moreover, compared with familiar voice stimulation, the peak Oxy-Hb level of the DLPFC-L was lower under unfamiliar voice stimulation, further supporting this mechanism: the DLPFC shows weakened responses to low-salience stimuli, leading to reduced peak activation, which reflects both the generalized cognitive decline in DOC and the DLPFC's limited ability to mobilize neural resources to cope with novel inputs (45, 46).

The TC, as a key node of the SN, is responsible for emotional regulation, the integration of internal and external information, and auditory processing, including language comprehension and auditory network functions (45, 47).

Compared with familiar voice stimulation, the peak Oxy-Hb level of TC-L significantly increased under unfamiliar voice stimulation, which may be attributed to the salience detection and alerting functions of the SN.

In patients with DOC, unfamiliar voices, as novel stimuli, may be identified as highly uncertain events that require prioritization for threat evaluation or environmental monitoring, thereby enhancing neuronal activity (reflected by an elevated peak Oxy-Hb level).

In contrast, familiar voices, although salient because of their emotional or personal relevance (e.g., memory association), are relatively predictable and therefore may evoke a lower peak response despite being classified as high-salience inputs (46, 48–50).

As a central hub of the auditory network, the increased peak response of the TC-L suggests excessive mobilization of auditory processing resources when unfamiliar voices are dealt with, possibly as a compensatory mechanism for perceptual deficits associated with impaired consciousness.

Notably, the elevated peak Oxy-Hb level of the TC-L contrasts with the deactivation of the DLPFC-L, highlighting complementary interactions between networks: while the TC-L upregulates the processing of novel inputs, the DLPFC-L downregulates activity to conserve resources (51, 52).

Thet PreM & SMC-L, as a subcomponent of the ECN, plays a dominant role in motor planning, action execution, and sequence coordination (53, 54). Compared with familiar voice stimulation, the initial slope of the ΔHbOC in PreM & SMC-L was smaller under unfamiliar voice stimulation, reflecting adaptive inhibition of motor preparation within the PreM & SMC-L (55, 56).

In states of impaired consciousness, unfamiliar voices may be perceived as potential threats or irrelevant inputs, triggering a delayed response mechanism in the PreM & SMC-L to suppress ineffective motor preparation (a smaller slope indicating delayed neural activation), thereby preventing unnecessary energy expenditure.

In contrast, familiar voices may rapidly activate the motor network through emotional relevance, facilitating behavioral preparation, as indicated by a steeper initial slope (27).

This mechanism is consistent with the functional division within the ECN, whereby the DLPFC-L is primarily responsible for cognitive control, whereas the PreM & SMC-L is dedicated to behavioral output (53).

In summary, in DOC patients, under UFV SON calling stimulation, the DLPFC exhibited deactivation and lower peak values, reflecting resource conservation, whereas under FV SON stimulation, it maintained activation. Conversely, under UFV SON calling stimulation, peak values of TC were higher, indicating salience detection and compensatory auditory mobilization, highlighting complementary interactions with the DLPFC. Furthermore, under UFV SON calling stimulation, the PreM & SMC-L showed a smaller initial slope, indicating adaptive inhibition of motor preparation, while under familiar voices, steeper slopes facilitated behavioral preparation.

This study further examined the associations between levels of consciousness, as indexed by CRS-*R* total and subscale scores, and fNIRS-based hemodynamic responses. Under the familiar voice condition, peak hemodynamic responses in the DLPFC-L and TC-L regions were positively associated with VFS scores, whereas the response velocity in the TC-L region was negatively associated with the CRS-*R* total score. In addition, peak activation in the PMC-L was negatively correlated with OFS and CS scores, and the response velocity in this region was negatively correlated with oromotor function.

In the unfamiliar voice condition, response velocity in the DLPFC-L region was positively associated with auditory function scores. The peak responses in the TC-L and Broca-L were positively correlated with the CRS-*R* total score, as well as the visual function and arousal subscale scores. Moreover, peak activation in the PMC-L was positively associated with the CRS-*R* total scores and the scores for the auditory function and visual function subscales.

Higher levels of consciousness were associated with slower activation velocity in the TC-L in response to familiar voices. Patients with better visual function exhibited stronger activation in the DLPFC-L and TC-L under familiar voice stimulation. Conversely, better oromotor (e.g., command-following vocalizations) or communication function (e.g., non-verbal responses) was associated with reduced activation in the PMC-L under familiar voices, and better oromotor function was also correlated with slower PMC-L activation. Under unfamiliar voice stimulation, higher overall CRS-*R* scores, better visual function, and greater arousal were associated with increased peak hemodynamic responses in the TC-L. Similarly, higher total CRS-*R* scores, auditory function, and visual function predicted greater peak responses in the PMC-L, whereas higher total CRS-*R* scores, visual function, and arousal predicted stronger peak responses in the left Broca area. Finally, better auditory function was associated with faster hemodynamic response velocity in the DLPFC-L under unfamiliar voice stimulation.

The DLPFC, a core node of the ECN, governs executive control and attentional allocation (56). In patients with disorders of consciousness, better visual function may support more effective executive control and attentional allocation, facilitating the association of familiar voices with visual representations, such as the caller's face. Under novel stimuli, patients with superior auditory function may more rapidly redirect attentional resources toward unexpected inputs (48). The TC, a node of the SN (45), shows enhanced alerting and monitoring responses to novel stimuli in patients with higher arousal and visual function, promoting wakefulness (49). Additionally, more efficient retrieval of autobiographical memory may facilitate cross-modal binding between auditory and visual information (57). Higher overall consciousness levels are also associated with more mature auditory processing, resulting in slower TC-L activation in response to familiar voices that engage autobiographical memory, which is consistent with predictive coding theory (46). The Broca area, which is involved in implicit speech processing, such as prosodic analysis (47), is more strongly activated in patients with greater arousal and visual function, supporting the maintenance of wakefulness and minimizing attentional drift (58). Enhanced language network function may also improve cross-modal attentional allocation between auditory and visual modalities (59). In patients with better oromotor function, the PMC-L exhibits reduced resource consumption and slower recruitment, indicating that automated oromotor outputs can be generated without strong activation, reflecting improved neural efficiency (54). In contrast, patients with superior visual and auditory function require stronger PMC-L recruitment to support orienting behaviors, such as auditory localization and visual search, suggesting that PMC-L activation serves as a key marker of attempts to interact with auditory–visual stimuli during recovery of consciousness (1, 60).

This study shows brain activation patterns during different SON calling stimulation paradigms. Under FV stimulation, higher visual function is related to stronger DLPFC and TC activation, reflecting better executive control and cross-modal integration, while higher degree of consciousness is linked to slower TC activation. Better oromotor function is related to reduced and slower PMC activation, indicating higher neural efficiency. Under FV stimulation, higher CRS-*R* scores, arousal, and better sensory function are associated with stronger responses in TC, Broca, and PMC, reflecting enhanced salience detection, arousal maintenance, and compensatory orienting processing. The findings of this study hold significant implications for the assessment and occupational therapy of patients with DOC. Under FV stimulation, the DLPFC remained activated, indicating that the patient was capable of mobilizing working memory and attention to process familiar information. Under UFV stimulation, the peak values of TC increased, demonstrating that the patient could still recognize novel stimuli and engage in environmental monitoring. This differentiated neural response to sounds of varying familiarity confirms that the patient not only retained basic auditory perception but also preserved the ability to interact between higher executive control and salience detection, which is a key feature distinguishing MCS from lower consciousness states (61). The differences in brain region activation patterns under varying stimuli can serve as objective indicators for evaluating patients' residual cognitive and motor preparation potential, thus addressing the limitations of traditional behavioral assessments, which are prone to subjective influence. The steeper slope exhibited by PreM & SMC under FV stimulation (behavioral preparation) and the sustained activation of the DLPFC help in identifying patients with cognitive motor dissociation, thereby avoiding misdiagnosis (5). The peak values in Broca's area under UFV stimulation not only serves as an objective indicator of the preserved language comprehension networks but also reflects patients' processing capacity for novel linguistic information and their neural readiness for verbal output. This finding may serve as a valuable neuroimaging biomarker for predicting subsequent language function recovery in patients with DOC (62). Since FV stimulation effectively maintain DLPFC activation and promote behavioral readiness in the PreM & SMC, while UFV stimulation tend to preserve cognitive resources and inhibit motor responses, rehabilitation training should prioritize using FV stimulation from relatives as auditory stimuli to more effectively engage patients' cognitive resources and elicit motor responses. However, due to limitations in sample size and study design, this aspect has not been explored in the current study. Further research can be conducted.

Despite these promising findings, several limitations of this study must be acknowledged. First, participant recruitment was conducted at a single center, which may limit the generalizability of the findings to broader populations. Second, this study is a descriptive investigation and, therefore, does not allow conclusions regarding the long-term effects of auditory stimulation interventions on the brains of patients with disorders of consciousness. Additionally, the sample size included in this study is relatively small, and no further subgroup stratification analysis was performed on the basis of the specific DOC types, which may affect the statistical power and specificity of the study results; moreover, the small sample size also limits the ability to consider potential confounding factors (such as the duration of disease, age, and sex). Moreover, this study did not quantify whole-brain effective connectivity and global information broadcasting capacity. Finally, previous studies have primarily used EEG or fMRI to investigate brain activity or structural changes in patients with disorders of consciousness under real-time or long-term auditory stimulation. However, the temporal and spatial resolutions of EEG and fMRI differ fundamentally from those of fNIRS, and prior work has not systematically examined the effect of stimulus familiarity on auditory processing. Therefore, the discussion does not directly compare the present findings with those of earlier studies. Future research should expand sample sizes and conduct multicenter clinical trials to further characterize patients across different types of disorders of consciousness and to investigate the influence of factors such as disease duration and sex. The combination of complementary computational approaches such as Multivariate Ornstein-Uhlenbeck Effective Connectivity and Ignition or related whole-brain dynamic modeling frameworks with the present method will help to further reveal the mechanisms underlying neurological functional recovery, which is also highly valuable (63). Moreover, combining fNIRS with other electrophysiological or neuroimaging modalities in multimodal studies could provide a deeper understanding of functional brain characteristics in these patients, ultimately informing the development of personalized rehabilitation protocols, efficacy evaluation, and prognostic assessment (64).

## 5 Conclusion

In conclusion, DOC patients show distinct cortical activation patterns under familiar vs. unfamiliar SON calling stimulation. This study is helpful for guiding auditory stimulation strategies or personalized rehabilitation in DOC. These conclusions warrant further validation in future larger, multicenter studies, multimodal neuroimaging, studies, and intervention-based studies.

## Data Availability

The raw data supporting the conclusions of this article will be made available by the authors, without undue reservation.
